# Minimally invasive percutaneous plate osteosynthesis versus intramedullary nail fixation for closed distal tibial fractures: a meta-analysis of the clinical outcomes

**DOI:** 10.1051/sicotj/2018055

**Published:** 2018-12-18

**Authors:** En Lin Goh, Swathikan Chidambaram, Denise Eigenmann, Shaocheng Ma, Gareth G. Jones

**Affiliations:** 1 Faculty of Medicine, Imperial College London, South Kensington Campus, London SW7 2AZ UK; 2 Department of General Surgery, Cantonal Hospital Lucerne, Spitalstrasse, 6000 Lucerne Switzerland; 3 Biomechanics Research Group, Imperial College London, South Kensington Campus, 774, 7th Floor, City and Guilds Building, London SW7 2AZ UK; 4 MSK Laboratory, Department of Surgery and Cancer, Faculty of Medicine, Imperial College London, London W6 8PR UK

**Keywords:** Distal Tibial Fractures, Plate, Intramedullary Nail, Meta-Analysis, Clinical Outcomes.

## Abstract

*Introduction*: Minimally invasive percutaneous plate osteosynthesis (MIPPO) has emerged as a viable alternative for the treatment of distal tibial fractures. However, the clinical outcomes of this procedure compared to intramedullary (IM) nail fixation have yet to be established. The present meta-analysis aims to compare the clinical outcomes following MIPPO and IM nail fixation for closed distal tibial fractures.

*Methods*: MEDLINE and EMBASE databases were searched from date of inception to 10th April 2017. Randomized controlled trials (RCTs) comparing MIPPO with IM nail fixation for closed and Gustilo Grade I distal tibial fractures were included. Outcomes assessed included time to union, complications and functional outcomes. Quality and risk of bias of the RCTs were assessed using the Cochrane Collaboration Tool.

*Results*: Five RCTs comprising 497 patients were included. MIPPO was associated with a longer time to union (MD: 1.08, 95% CI: 0.26, 1.90, *p *= 0.010, *I* ^2 ^= 84%) and increased risk of wound complications (RR: 1.58, 95% CI: 1.01, 2.46, *p *= 0.04, *I* ^2^ = 0%). Both MIPPO and IM nail fixation had comparable risks of malunion, delayed union, non-union and deep infections, with similar functional outcomes.

*Discussion*: Compared to IM nail fixation, a MIPPO fixation technique for distal tibial fractures is associated with a longer time to fracture union and an increased risk of wound complications. Neither technique demonstrates a clear advantage with regard to risk of malunion/non-union, or functional outcome. Assuming equivalent surgical expertise with both techniques, the results suggest that IM nail fixation is the treatment modality of choice for these challenging fractures.

## Introduction

Distal tibial fractures are common injuries affecting individuals of all ages, with an incidence of up to 28 per 10,000 individuals per year [1]. These fractures require admission to hospital and are associated with a significant economic burden [2]. Cost-analyses suggest that these fractures incur an additional $15,000 per individual due to the increased risk of complications [3,4]. Surgical intervention is usually required to realign the fracture, support local tissues and permit mobility of the adjacent joints. The management of these fractures remains difficult due to several factors; notably, the limited soft tissue coverage, poor vascularity of the region and proximity to the ankle joint [5,6]. Furthermore, these patients are at risk of developing infection, delayed union, non-union and malunion [7].

Traditional open reduction and internal fixation with a plate and screws is associated with a high risk of infection, wound breakdown and delayed union due to the required soft tissue dissection [8]. This has led to the increasing use of more soft tissue preserving treatment options such as minimally invasive percutaneous plate osteosynthesis (MIPPO) and intramedullary (IM) nail fixation. However, it is unclear which of these techniques is preferable; IM nailing is associated with an increased risk of malalignment and knee pain, while MIPPO is technically challenging, involves more soft tissue dissection and often requires metal work removal following fracture union [6].

Previous meta-analyses comparing MIPPO and IM nail fixation have been limited by small sample sizes, significant heterogeneity and low quality studies [7,9,10]. Even accepting these limitations, the inclusion of all grades of open fractures in these studies makes it difficult to use the results to inform clinical practice. The present meta-analysis aims to address these concerns by comparing the clinical outcomes following MIPPO and IM nail fixation for closed and Gustilo Grade I distal tibial fractures using data only from randomized controlled trials (RCTs) [11,12].

## Materials and methods

Literature search methods, inclusion and exclusion criteria, outcome measures and statistical analysis were defined according to the Preferred Reporting Items for Systematic Reviews and Meta-Analyses (PRISMA) [13]. Patients were not involved in the conception, design, analysis, drafting, interpretation or revision of this research. Thus, ethics approval was not required.

### Electronic search

The following databases were searched: (a) MEDLINE (1946 till second week of April of 2018) via OvidSP, last search on 10th April 2018; (b) MEDLINE in-process and other non-indexed citations (latest issue) via OvidSP, last search on 10th April 2018; (c) Ovid EMBASE (1974 to latest issue), last search 10th April 2018; (d) Scopus (1996 till present), last search on 10th April 2018. Search terms used three strings, which were then linked by an AND modifier. The first string included tibial fractures OR fractures of the tibia, the second string included intramedullary nail OR bone nail and the third string included plate-and-screw OR plate OR bone plate. Truncated search terms utilizing the wildcard character and the “related articles” function were used to broaden the search. Additionally, the references of included articles were hand-searched to identify any additional studies.

### Study selection

All RCTs in which MIPPO was compared with IM nail fixation were selected. In addition, all studies included in the meta-analysis met the following criteria: (a) individuals were 16 years or older, (b) closed or Gustilo Grade I acute, displaced extra-articular fractures of the distal tibia, (c) fracture does not extend into ankle joint, (d) no history of total knee arthroplasty, deformity of the tibia, congenital abnormality, neuromuscular disease or chronic inflammatory joint disease, (e) no contraindication to anesthesia, (f) no evidence of polytrauma, (g) article was published or accepted for publication as full-length articles. No restrictions were made on language. Non-human studies, experimental trials, case-control studies, cohort studies, review articles, editorials, case reports, letters, conference abstracts and unpublished studies were excluded.

### Outcome measures

Outcomes assessed were time to union, complications (delayed union [radiographic union >24 weeks], non-union [failed union by 36 weeks], malunion [varus or valgus deformity >5°, anterior/posterior angulation >10°, rotational deformity >10°, and shortening >10 mm] as assessed using computed radiography, wound and deep infections) and functional outcomes (Disability Rating Index [DRI] score, Oleureud and Moleander Ankle Score [OMAS], EuroQol Health-Related Quality-of-Life 3-Level [EuroQOL-5D-3L] index score, American Orthopaedic Foot and Ankle Surgery [AOFAS] score, Foot Function Index [FFI], Johner and Wruh's criteria).

### Data extraction

Two independent reviewers (E.L.G and S.C.) screened all the titles and abstracts for inclusion, both of whom were blinded to authors, journals, institutional affiliations and dates of publication. Both reviewers evaluated each selected reference independently and summarized relevant study characteristics. In case of disagreement, a consensual decision between the two reviewers under involvement of a third independent reviewer (S.M.) was reached. The following data items were extracted: the year of publication, study design, sample size, country of study, type of patients, patient characteristics, outcome measures and conclusions. Authors of the original publications would be contacted in the event of insufficient data, but this was not necessary. Data were entered into Review Manager 5.3 (Cochrane Collaboration, Oxford, United Kingdom).

### Quality assessment and risk of bias

The quality and risk of bias of the RCTs was assessed using the Cochrane Collaboration Tool. All risk of bias domains were given equal consideration. For sensitivity analysis, trials were defined as having an overall high risk of bias if they were assessed to be of high risk in any of the following domains: random sequence generation, allocation concealment, outcome data incomplete or selective reporting. Quality of evidence was assessed using the Grading of Recommendations Assessment, Development and Evaluation (GRADE) Working Group grades of evidence.

### Statistical analysis

Risk ratio (RR) and mean difference (MD) are presented with 95% confidence interval (CI). Review Manager 5.3 (Cochrane Collaboration, Oxford, United Kingdom) was used for data analysis. Medians were converted to means using the formula described by Hozo et al. [14]. The fixed-effects model was used to pool the results. The standard heterogeneity test, the *I* ^2^ statistic, was used to assess the consistency of the effect sizes, which indicates the percentage of the variability in effect estimates because of true between-study variance rather than within-study variance. Statistical heterogeneity was defined as low, moderate and high with an *I* ^2^ of above 25%, 50% and 75%, respectively [15]. Results above 60% were considered as substantial heterogeneity. The risk of publication bias was assessed using a funnel plot.

## Results

### Study characteristics

**Fig. 1:**
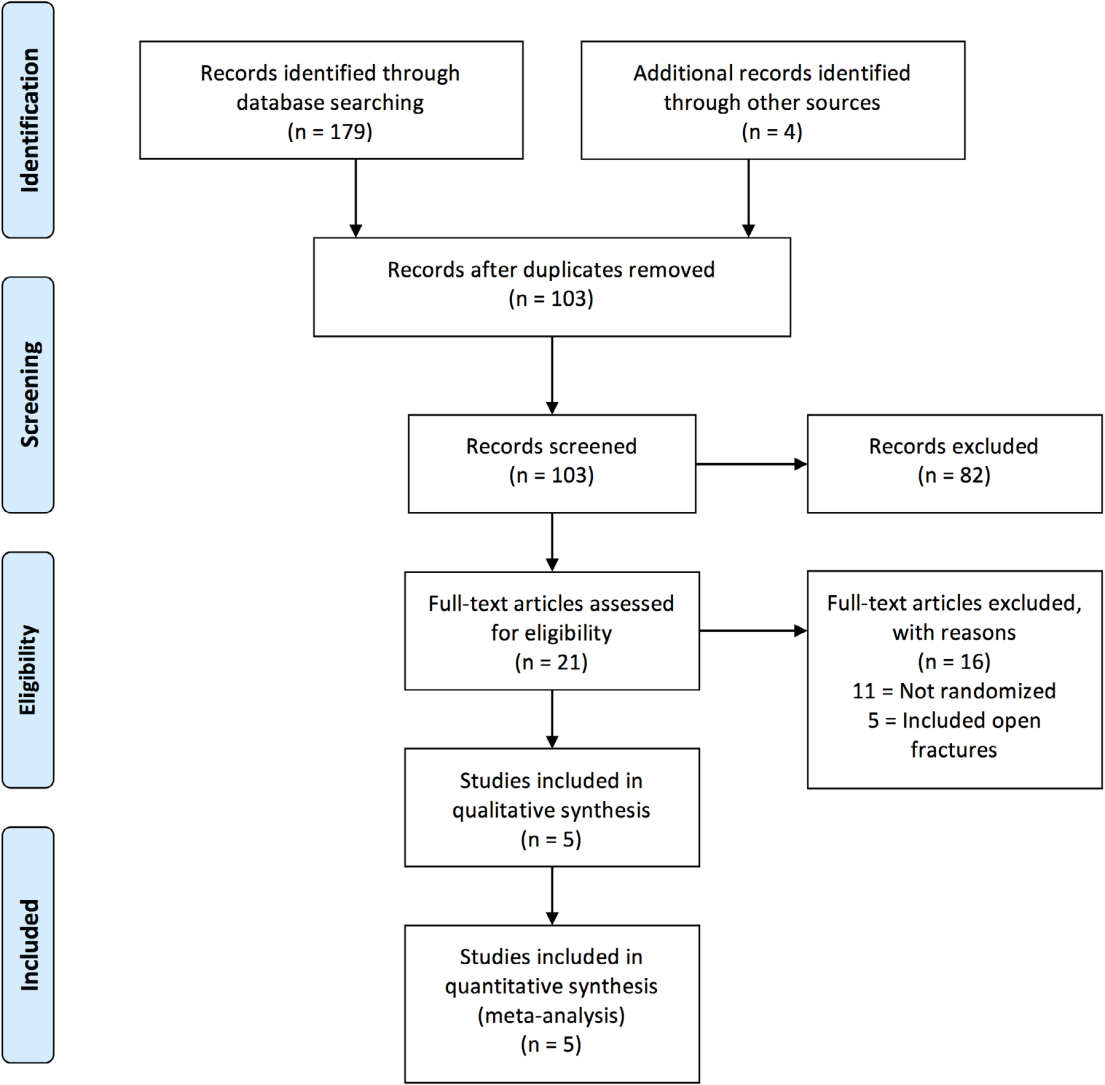
PRISMA flow chart.

Five RCTs comprising of 497 patients were included in this meta-analysis (Figure 1, Table 1) [5,16–19]. There were 248 patients in the IM nail fixation group and 249 patients in the MIPPO group. The total number of patients in each study ranged from 24 to 321. Two studies were conducted in the United Kingdom, one study in Turkey, one study in China and one study in India. Fractures included in the studies ranged from closed to Gustilo Grade I fractures. Only closed and Gustilo Grade I fractures of the distal tibia were included (AO classification 43-A) [11]. Males comprised 62.6% of the total study population. Follow-up across all studies was at least 12 months.

**Table 1:** Study characteristics.

Study	Country	Number of patients		Age (years)		Gender (M/F)	Follow-up (months)	Loss to follow-up (IM nail/plate)	AO classification		Fracture type
								
		IM nail	Plate	IM nail	Plate				IM nail	Plate	
Guo et al. (2010)	China	44	41	44.2 (27–70)	44.4 (23–69)	50/35	12	13/13	43-A1: 13 43-A2: 16 43-A3: 15	43-A1: 13 43-A2: 12 43-A3: 16	Closed or Gustilo I
Mauffrey et al. (2012)	UK	12	12	50 (39–60)	33 (24–43)	16/8	12	1/1	–	–	Closed or Gustilo I
Polat et al. (2015)	Turkey	10	15	34.0 (9.7)	36.4 (10.7)	16/9	23.1	0/0	–	–	Closed
Daolagupu et al. (2017)	India	21	21	35.2 (9.2)	39.1 (10.1)	32/10	12	0/0	43-A1: 11 43-A2: 6 43-A3: 4	43-A1: 10 43-A2: 9 43-A3: 2	Closed
Costa et al. (2017)	UK	161	160	44.3 (16.3)	45.8 (16.3)	197/124	12	18/19	–	–	Closed

### Quality assessment and risk of bias of included studies

**Fig. 2:**
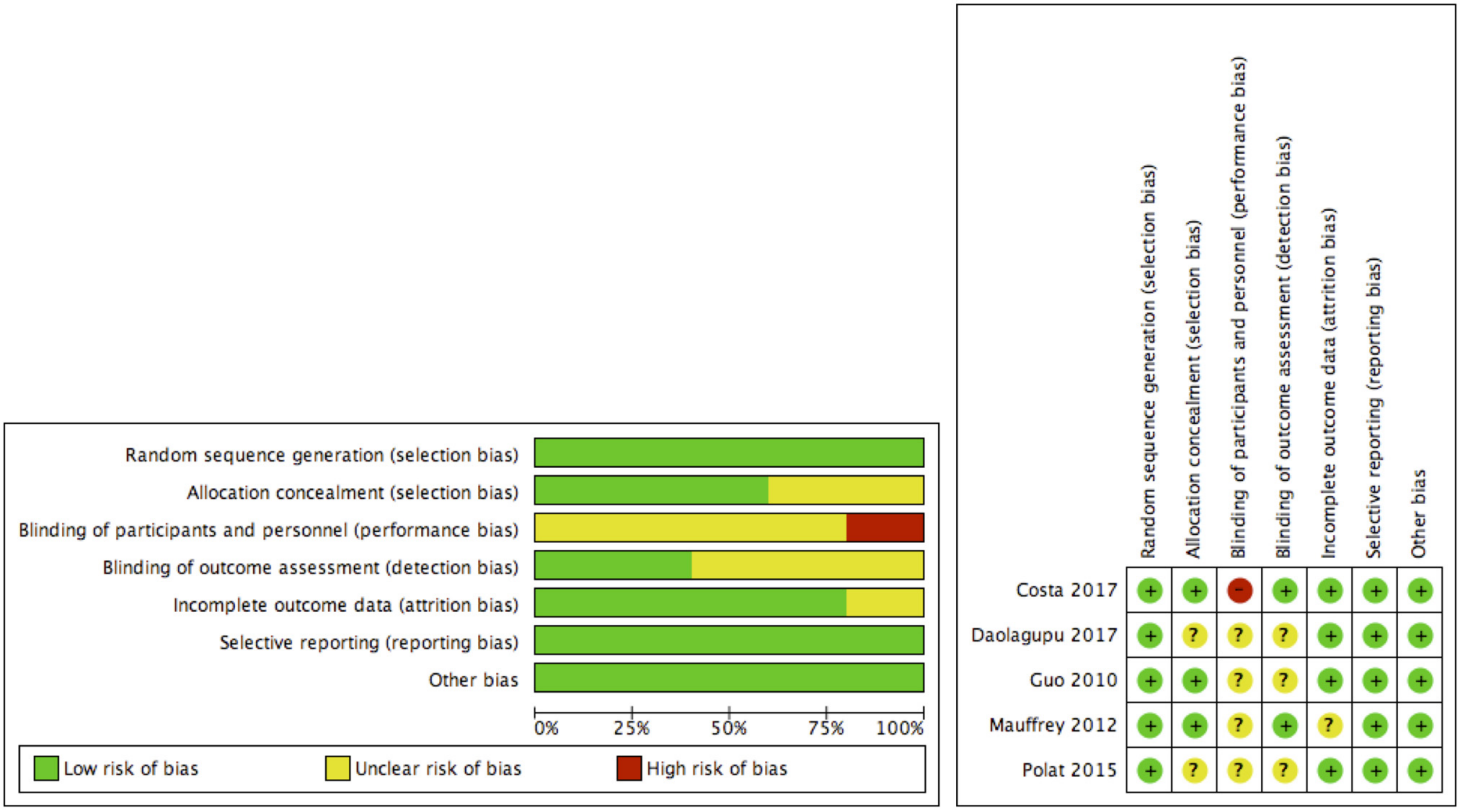
Risk of bias tables.

**Fig. 3:**
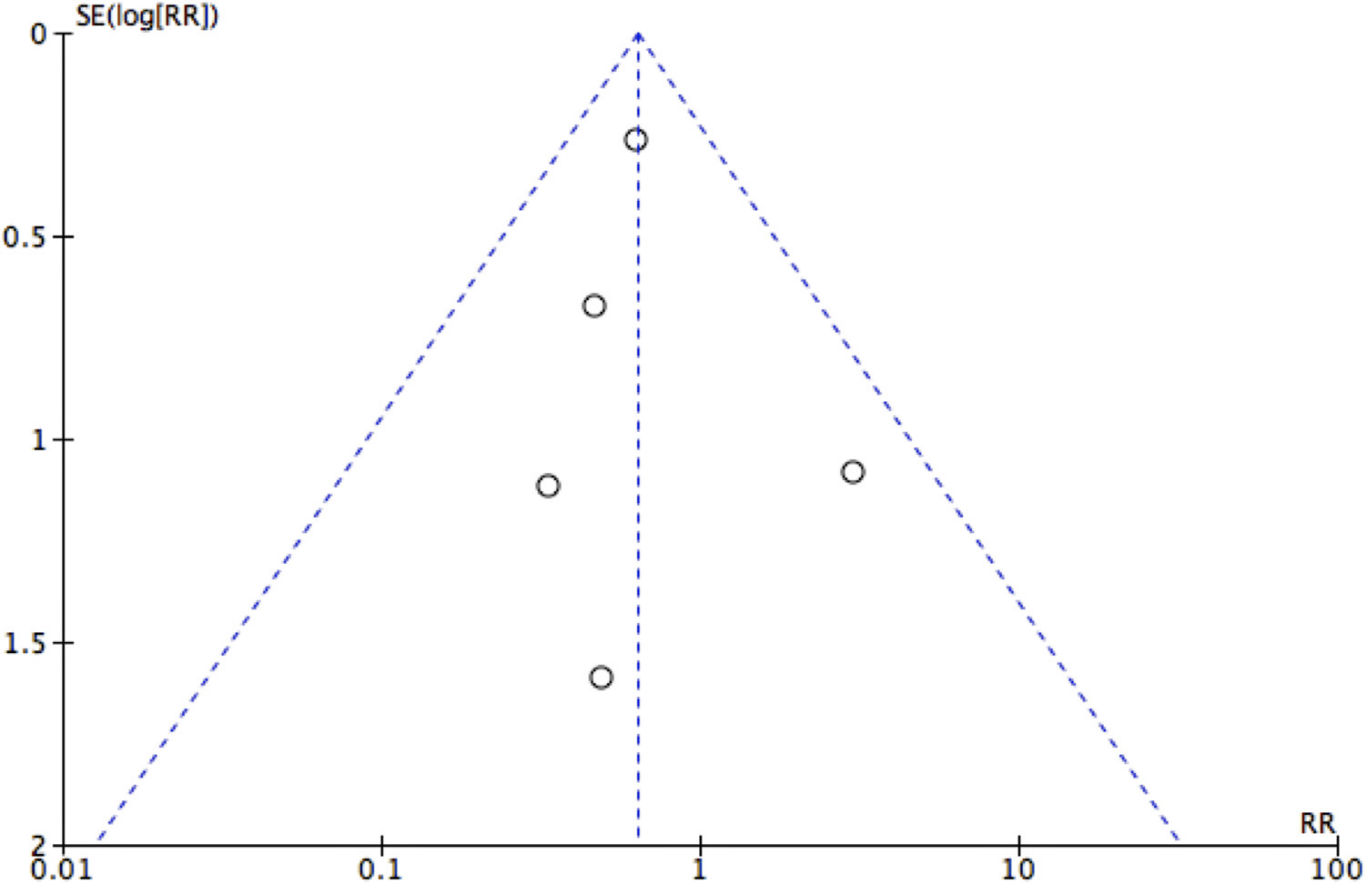
Funnel plot assessing publication bias.

Most studies were found to exhibit a moderate risk of bias (Figure 2). Overall, there was low risk of bias in terms of random sequence generation, allocation concealment, incomplete outcome data and selective reporting. All but one study failed to provide information regarding blinding of participants. Meanwhile, only two studies provided information on whether the assessors were blinded. The risk of publication bias was examined using a funnel plot (Figure 3). No asymmetry was detected.

### Time to union

**Fig. 4:**
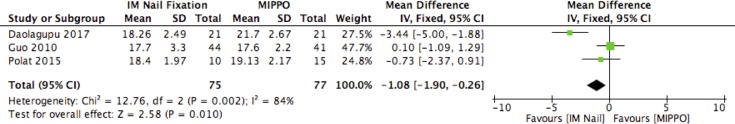
Time to union.

Time to union was reported in three studies, with 152 patients (Figure 4). MIPPO was associated with a longer time to union, with a MD of 1.08 weeks (95% CI: 0.26, 1.90, *p *= 0.010, *I* ^2^ = 84%). Substantial heterogeneity was present in the data and the evidence was deemed to be of low quality.

### Complication rates

**Fig. 5:**
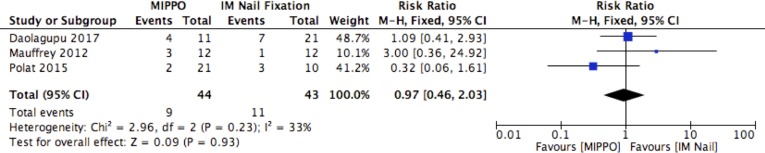
Union complications.

Complications associated with union, including delayed union, non-union and malunion were reported in three studies comprising 87 patients (Figure 5). Overall, complication rates were comparable between MIPPO and IM nail fixation with a RR of 0.97 (95% CI: 0.46, 2.03, *p *= 0.93, *I* ^2^ = 33%). Further analysis revealed a lower risk of malunion associated with MIPPO, with a RR of 0.50 (95% CI: 0.19, 1.32, *p *= 0.16, *I* ^2^ = 0%). Low heterogeneity was present in the data and the evidence was deemed to be of moderate quality. Wound complication rates were reported in five studies with a total of 490 patients. MIPPO had a higher risk of wound complications with a RR of 1.58 (95% CI: 1.01, 2.46, *p *= 0.04, *I* ^2^ = 0%) in favour of IM nail fixation. No heterogeneity was present in the data and the evidence was deemed to be of high quality. The rates of deep infections were reported in five studies comprising of 412 patients. There was a higher rate of deep infections following MIPPO, with a RR of 3.57 (95% CI: 0.91, 13.95, *p *= 0.07, *I* ^2^ = 0%). No heterogeneity was present in the data and the evidence was deemed to be of moderate quality.

**Fig. 6:**
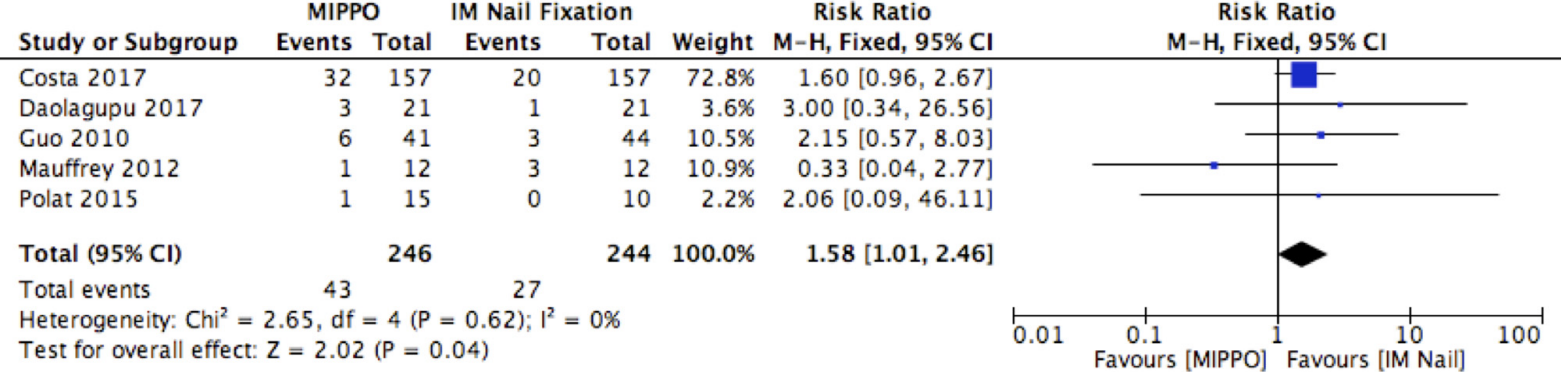
Wound complications.

**Fig. 7:**
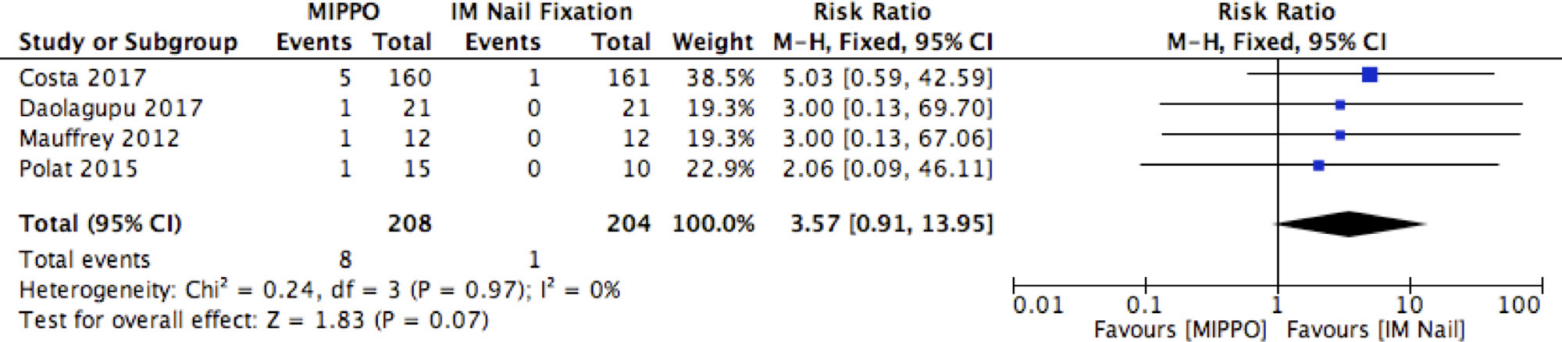
Deep infections.

### Functional outcomes

Functional outcomes reported include the DRI score, OMAS, EuroQOL-5D-3L, AOFAS score, FFI and Johner and Wruh's criteria. The DRI score, OMAS and EuroQOL-5D-3L were reported in two studies of 282 patients [5,17]. The AOFAS score was reported in two studies comprising 153 patients [18,19]. The FFI was reported in one study of 25 patients [16]. The Johner and Wruh's criteria was reported in another study of 42 patients [19]. MIPPO was associated with a higher DRI score, with a MD of 7.84 (95% CI: 3.51, 12.16, *p *= 0.0004, *I* ^2^ = 0%), 4.34 (95% CI: −0.97, 9.64, *p *= 0.11, *I* ^2^ = 0%) and 1.35 (95% CI: −4.21, 6.91, *p *= 0.63, *I* ^2^ = 0%) at 3, 6 and 12 months. MIPPO was also associated with a lower OMAS, with a MD of −6.42 (95% CI: −10.61, −2.23 *p *= 0.003, *I* ^2^ = 0%), −5.22 (95% CI: −10.51, −0.06, *p *= 0.05, *I* ^2^ = 0%) and −4.54 (95% CI: −9.87, 0.80, *p *= 0.10, *I* ^2^ = 65%) at 3, 6 and 12 months, respectively. The EuroQOL-5D-3L was comparable between MIPPO and IM nail fixation, with a MD of −0.04 (95% CI: −0.10, 0.01, *p *= 0.13, *I* ^2^ = 0%), −0.06 (95% CI: −0.12, −0.01, *p *= 0.03, *I* ^2^ = 22%) and −0.02 (95% CI: −0.08, 0.03, *p *= 0.38, *I* ^2^ = 82%) at 3, 6 and 12 months, respectively.

## Discussion

This meta-analysis set out to compare MIPPO and IM nailing of closed and Gustilo Grade I distal tibial fractures. It has identified a longer time to union, and increased rate of wound complications, with use of a MIPPO fixation technique. The overall rate of delayed union, non-union and malunion was comparable between both groups. In terms of functional outcome, both groups demonstrated comparable DRI, OMAS and EuroQOL-5D-3L index scores at 12 months post-operatively.

Contrary to the findings of previous studies, IM nailing does not appear to be associated with a higher risk of malunion [6,8,20–22]. This might be because the surgeons included in the RCTs were high volume and experienced trauma surgeons, although unfortunately this variable was not accounted for in any of the included studies [8,17,20,21,23]. This can be explained by accurate fracture reduction in the included studies, which can be achieved with the judicious use of intra-operative reduction aids and biplanar imaging and may reflect the level of surgical experience and operative technique, both of which were not accounted for in any of the previous studies. In the included studies, anatomical reduction confirmed with intra-operative imaging was achieved in all cases, although the reduction technique was left at the discretion of the operating surgeon. External reduction clamps were used to achieve this in the event of poor reduction quality initially. Furthermore, this suggests that accurate fracture reduction through the judicious use of intra-operative reduction aids and biplanar imaging might be a more important factor in preventing malunion than loss of correction post-operatively due to poor screw fixation in the distal metaphyseal bone [24].

There was a comparable risk of delayed union and non-union following MIPPO and IM nail fixation. Previous studies have demonstrated a lower risk of delayed union and non-union with MIPPO compared to IM nail fixation but these studies have methodological concerns; in particular, they are highly susceptible to bias and confounding factors [25,26]. Pooled results in a previous meta-analysis revealed a marginally lower risk of delayed union with MIPPO but the evidence was deemed to be of low quality due to the inclusion of cohort studies [9]. The present meta-analysis was restricted to RCTs, which may explain the difference in results.

MIPPO was associated with a higher risk of wound complications compared to IM nail fixation, which corroborates previous work on this topic [10]. Whilst MIPPO requires smaller incisions and causes less iatrogenic soft tissue damage than traditional open reduction and internal fixation, it remains more locally invasive than IM nailing techniques. It should be noted that the rate of deep infections was comparable between the two groups.

There are limitations in our analysis. Most of the included RCTs had a small patient population and may therefore be underpowered to detect differences across all outcomes, especially functional ones. Gustilo Grade I fractures were included in two studies and assumed to behave the same as closed fractures. In addition, the complexity of the fracture pattern (43-A1, A2 or A3) was not considered, which may affect results. For instance, Guo et al. included a larger number of 43-A3 fractures in their study compared to Daolagupu et al., which could explain the differences in time to union between IM nail fixation and MIPPO in both studies. However, larger-scale analysis of both procedures in these fracture subtypes will be necessary to draw a firm conclusion. Blinding of both surgeons and patients was not possible, with the surgical scars indicating to the patient which type of fixation device was employed. Thus, this could introduce an element of response bias into the assessment of functional outcomes although radiographic outcomes are less likely to have been influenced. Additionally, different surgical techniques and devices were used in each study, which may impact on the efficacy of treatment and complication rates. As an example, fibula fixation was performed in some studies at the discretion of the operating surgeon: a technique which might reduce the risk of malunion but has also been shown to increase the risk of delayed union and non-union [16,17,27,28]. Furthermore, different functional outcome scores were used, which precluded any form of pooled analysis. Although the overall EuroQOL-5D-3L index score was reported, scores for the individual dimensions were not available, so it is possible that patients treated with IM nailing and MIPPO could have scored differently in each dimension, despite having a similar overall score.

## Conclusion

This meta-analysis presents high-quality evidence demonstrating a higher rate of wound complications and low-quality evidence showing longer time to union with MIPPO. There is moderate quality evidence supporting comparable rates of delayed union, non-union, malunion and deep infections but only very low quality evidence with regard to functional outcomes. In conclusion, IM nail fixation demonstrates advantages over a MIPPO technique, and assuming equivalent surgical experience should be the recommended technique for distal tibial fractures. Future RCTs should address the issue of surgical experience on outcome.

## Conflict of interest

The authors declare that they have no conflict of interest and received no funding for this work.
